# Impact of Silver-Decorated Graphene Oxide (Ag-GO) towards Improving the Characteristics of Nanohybrid Polysulfone Membranes

**DOI:** 10.3390/membranes13060602

**Published:** 2023-06-15

**Authors:** Nur Syahirah Suhalim, Norherdawati Kasim, Ebrahim Mahmoudi, Intan Juliana Shamsudin, Nor Laili-Azua Jamari, Fathiah Mohamed Zuki

**Affiliations:** 1 Faculty of Defence Science and Technology, National Defence University of Malaysia, Kem Sungai Besi, Kuala Lumpur 57000, Malaysia; 3201357@alfateh.upnm.edu.my; 2 Department of Chemistry & Biology, Centre for Defence Foundation Studies, National Defence University of Malaysia, Kem Sungai Besi, Kuala Lumpur 57000, Malaysia; intanjuliana@upnm.edu.my (I.J.S.); azua@upnm.edu.my (N.L.-A.J.); 3 Department of Chemical and Process Engineering, Faculty of Engineering and Built Environment, Universiti Kebangsaan Malaysia, Bangi 43600, Malaysia; mahmoudi.ebi@ukm.edu.my; 4 Department of Chemical Engineering, Faculty of Engineering, University of Malaya, Kuala Lumpur 50603, Malaysia; fathiahmz@um.edu.my

**Keywords:** mixed-matrix membranes, nanofiltration membrane, Donnan exclusion, iron removal, membrane hydrophilicity

## Abstract

The utilization of membranes has been extensively employed in the treatment of water and wastewater. Membrane fouling, attributed to the hydrophobic nature of membranes, constitutes a noteworthy concern in the realm of membrane separation. The mitigation of fouling can be achieved through the modification of membrane characteristics, including but not limited to hydrophilicity, morphology, and selectivity. In this study, a nanohybrid polysulfone (PSf) membrane embedded with silver–graphene oxide (Ag-GO) was fabricated to overcome problems related to biofouling. The embedment of Ag-GO nanoparticles (NPs) is the aim towards producing membranes with antimicrobial properties. The fabricated membranes at different compositions of NPs (0 wt%, 0.3 wt%, 0.5 wt%, and 0.8 wt%) are denoted as M0, M1, M2, and M3, respectively. These PSf/Ag-GO membranes were characterized using FTIR, water contact angle (WCA) goniometer, FESEM, and salt rejection. The additions of GO significantly improved the hydrophilicity of PSf membranes. An additional OH peak at 3380.84 cm^−1^ of the nanohybrid membrane from FTIR spectra may be related to hydroxyl (-OH) groups of GO. The WCA of the fabricated membranes decreased from 69.92° to 54.71°, which confirmed the improvement in its hydrophilicity. In comparison to the pure PSf membrane, the morphology of the finger-like structure of the fabricated nanohybrid membrane slightly bent with a larger bottom part. Among the fabricated membranes, M2 achieved the highest iron (Fe) removal, up to 93%. This finding proved that the addition of 0.5 wt% Ag-GO NPs enhanced the membrane water permeability together with its performance of ionic solute removal (Fe^2+^) from synthetic groundwater. In conclusion, embedding a small amount of Ag-GO NPs successfully improved the hydrophilicity of PSf membranes and was able to achieve high removal of Fe at 10–100 mg L^−1^ towards purification of groundwater for safe drinking water.

## 1. Introduction

Groundwater quality is currently being threatened by contaminants, including heavy metals, originating from both natural and anthropogenic sources [1,2]. Heavy metals are metallic elements with a density greater than that of water [3] and are known to have harmful effects on both the ecosystem and living organisms [4]. Despite this, it is important to note that heavy metals, including iron (Fe), manganese (Mn), zinc (Zn), copper (Cu), and nickel (Ni), play a vital role in the growth and development of both animals and plants, as long as they are present in limited quantities [5]. Fe and Mn are prevalent contaminants detected in groundwater, originating from both natural sources, such as soil and rocks, as well as anthropogenic activities, such as industrial wastewater discharge and excessive groundwater extraction [6,7]. At elevated concentrations, the presence of Fe and Mn in water can result in undesirable sensory attributes, such as taste, odor, and coloration [8]. Crittenden et al. [9] explored various treatment technologies to address this issue, ranging from membrane filtration, reverse osmosis, disinfection, granular filtration, gravity separation, aeration, and ion exchange to adsorption. The employment of membrane filtration technology has gained significant interest due to its numerous benefits, including minimal energy usage and the absence of chemical supplements [10].

Synthetic polymers are commonly utilized as the primary constituent of membranes, although alternative materials, such as metals and ceramics, can also be employed for membrane fabrication [11]. The materials frequently utilized in this context include polysulfone (PSf) [12], polypropylene (PP) [13], polyvinyl chloride (PVC) [14], polyamide (PA) [15,16,17], polyethersulfone (PES) [18], and polyvinylidene fluoride (PVDF) [19]. However, membrane technology has numerous drawbacks, including low water flux, poor rejection, and membrane fouling [20]. A superior membrane is characterized by its favorable conductivity properties, cost effectiveness, elevated membrane permeability and stability, exceptional mechanical features, and optimal water retention [21]. Among these polymers, polysulfone (PSf) is commonly utilized in membrane production due to its remarkable mechanical qualities, broad pH range for operation, thermal endurance, and durability against chemicals [22]. Nevertheless, there are some drawbacks of PSf due to its hydrophobic surfaces that lead to low permeability and high fouling [23]. There have been several attempts to alter or adjust the membrane permeability and hydrophobicity, including the development of composite membranes via plasma treatment [18], inter-facial polymerization [24,25,26], layer-by-layer deposition [27], UV-initiated grafting [28], and the incorporation of nanoparticles or antifouling agents [29]. Furthermore, many studies have shown that combining a membrane polymer with nanoparticles (NPs) can significantly improve the membrane performance [30]. Nanoparticles are commonly characterized as small particles possessing a dimension of less than 100 nm [31]. Nanoparticles that are commonly used for fabricating mixed-matrix membranes are iron oxide (Fe_3_O_4_) [32], zinc oxide [33], titanium dioxide (TiO_2_) [34], silver (Ag) [35], and graphene oxide (GO) [36].

Graphene oxide (GO) has garnered significant attention in the scientific community owing to its exceptional properties as a nanoparticle. The GO material exhibits a hexagonal carbon structure that is comparable to graphene. However, it also comprises various oxygen-based functional groups, such as alkoxy (C-O-C), hydroxyl (-OH), carboxylic acid (-COOH), and carbonyl (C=O) [37]. According to Smith et al. [38], GO employs a straightforward top-down synthesis approach. For instance, to investigate the preparation of reduced GO, Cao and Zhang [39] employed Hummers’ method. Zaaba et al. [40] employed a modified version of the Hummers’ method to synthesize graphene oxide (GO). Ma et al. [41] stated that by incorporating graphene oxide–polyethylene glycol with a PVDF nanocomposite, the hydrophilicity of the membranes increased significantly. The membrane’s hydrophilicity was enhanced due to the existence of oxygen-based functional groups. Upon dispersion of graphene in water, the carboxyl group undergoes hydrolysis, leading to the production of negatively charged acid and hydrogen ions. This process ultimately leads to the formation of stable oxidized graphene dispersion [42]. Aside from GO, Ag NPs have also risen in popularity due to their antibacterial properties. To develop antibacterial and antifouling properties, Zhu and Lua [43] employed interfacial polymerization to bind Ag NPs on the surface of an industrial PES membrane. Sprick [44] also developed a method for chemically immobilizing Ag NPs onto membranes without compromising the antimicrobial properties of the Ag NPs.

The synergetic effect of GO and Ag NPs offers distinctive characteristics, such as exceptional resistance to biofouling, hydrophilicity, elevated water flux, and good mechanical properties. Building upon these strengths, many researchers have decorated GO with Ag NPs and embedded it onto a different polymeric membrane, achieving excellent results [45,46]. This study aims to produce a nanohybrid polysulfone membrane embedded with silver-decorated graphene oxide (PSf/Ag-GO) to create an outstanding membrane. Specifically, this research investigates the impact of silver–graphene oxide towards the morphology and hydrophilicity of the nanohybrid membrane and evaluates its selectivity in terms of ionic solute removal. By focusing on these aspects, this study contributes to the advancement of nanohybrid membrane technology, which can lead to enhanced performance in diverse applications, including energy production and water treatment. The results of this study could help in designing and optimizing future nanohybrid membrane systems for more efficient and sustainable processes.

## 2. Materials and Methods

### 2.1. Materials

Ag-GO was synthesized in the Membrane Research Laboratory, Universiti Kebangsaan, Malaysia. Polysulfone (PSf) was obtained in pellet form from Sigma (Darmstadt, Germany). N-methyl-2-pyrrolidone (NMP) from R & M Chemicals (Kuala Lumpur, Malaysia) was used as the solvent, while ferrous chloride tetrahydrate (FeCl_2_H_8_O_4_) from Nacalai Tesque (Kyoto, Japan) was used to prepare artificial groundwater as feed solution.

### 2.2. Preparation of PSf/Ag-GO Membranes

The fabrication process of the PSf/Ag-GO membranes involved the use of the phase inversion method. The casting solution was created with PSf as the polymer and NMP as the solvent. The incorporation of Ag-GO NPs served as a hydrophilic modifier/additive, with the aim of enhancing the flux rate of the membrane. Different amounts of Ag-GO were added according to Table 1, whereby membranes M1, M2, and M3 consisted of 0.3%, 0.5%, and 0.8% of these NPs, respectively. Pure PSf membranes were prepared as control experiments.

The Ag-GO was initially dispersed in N-methyl-2-pyrrolidone (NMP) solvent and subjected to sonication for a duration of 30 min to achieve a uniform casting solution. Subsequently, the PSf was gradually introduced into the Ag-GO/NMP solution and stirred for a duration of 24 h. Next, the casting solution that had been prepared beforehand was applied onto a pristine glass plate and promptly submerged in deionized (DI) water for a duration of 15 s, following which it was detached from the glass plate. Then, the fabricated membranes were placed in a container containing deionized water.

### 2.3. Characterization of Membrane and Performance Test

**Figure 1:**
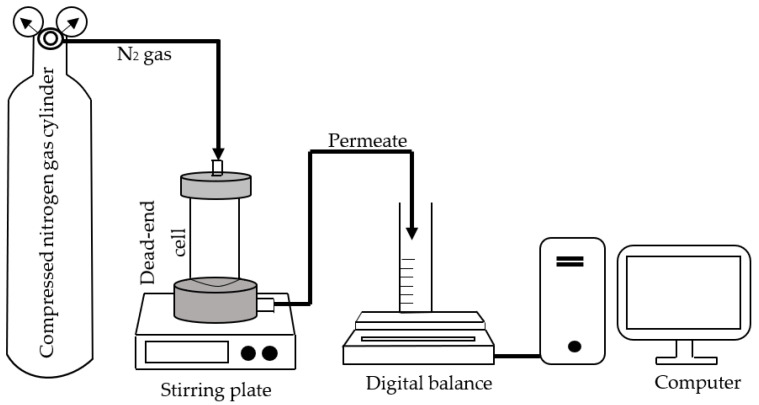
A bench-scale dead-end stirred cell filtration setup.

The functional groups present in the fabricated membranes were analyzed using a Frontier Fourier-transform infrared (FTIR) spectrometer manufactured by Perkin Elmer. The hydrophilicity of the membranes was evaluated through the utilization of a water contact angle goniometer (L2004A1, Os-sila, Leiden, The Netherlands) under ambient conditions. The cross-sectional structures of the fabricated membranes were examined using FESEM (Gemini SEM 500, Zeiss, Oberkochen, Germany). The membrane performance in terms of water permeability and removal capability was measured using a dead-end filtration nanofiltration system device, as depicted in Figure 1. The membrane water flux and salt rejection capacity were evaluated using a stirred cell methodology. Specifically, a flat sheet of the fabricated membrane coupon, which had an effective surface area of 14.6 cm^2^, was placed onto the bottom of the Sterlitech HP4750 stirred cell. The porous plate was utilized to provide support to the membrane, which was then subjected to compaction for a duration of 30 min, utilizing ultra-pure water. This was carried out to eliminate any residual chemicals and to achieve a uniform solution flux. Subsequently, the water flux was ascertained at varying levels of applied pressure, spanning from 5 to 9 bar.

The water flux was calculated using Equation (1):

(1) $$\mathrm{Water}\;\mathrm{flux},\;J=(\frac{∆V}{A∆t})$$

where *J* (L/m^2^ h) is the measured permeate flux, ∆*V* is the permeate cumulative volume (L), *A* is the effective membrane area (m^2^), and ∆*t* is the filtration time (h).

The observed rejection was calculated according to Equation (2):

(2) $$R=1-\frac{Cp}{Cf}\times 100$$

where *R* (%) is rejection of membrane, and *C*_p_ and *C*_f_ are concentration of permeate and feed solution (mg L^−1^), respectively.

## 3. Results and Discussion

### 3.1. Properties of PSf/Ag-GO Membranes

**Figure 2:**
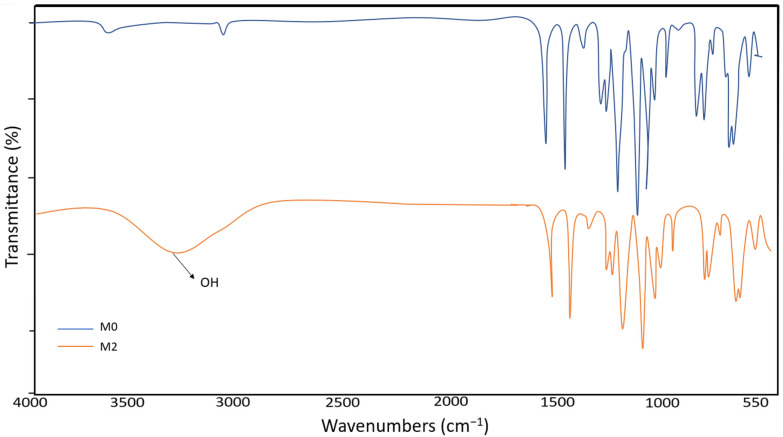
FTIR spectra of M0 and M2.

The fabricated membranes were analyzed for their functional groups using FTIR spectroscopy. In the present study, we performed characterization of all PSf membranes, whether fabricated with or without Ag-GO NPs. Figure 2 shows the FTIR spectra of the M0 and M2 membranes for comparative study, whereby both results could represent the M1 and M3 membranes for fabrication with NP embedment. The vibrational frequencies of peaks for membrane samples were determined and are listed in Table 2. From these results, there is an additional peak at 3380.84 cm^−1^ for the M2 membrane, which may be related to the hydroxyl (-OH) groups of GO (Mahmoudi, 2017) [47]. Other than that, the results show no distinct difference between the samples, which clearly implies that the addition of GO promoted the -OH group in the fabricated membranes, which tend to improve the hydrophobicity of polymeric membrane solutions. The similarity observed in the samples may be explained by the fact that the GO and PSf peaks overlap.

### 3.2. Properties of Composite PSf-GO Membranes

**Figure 3:**
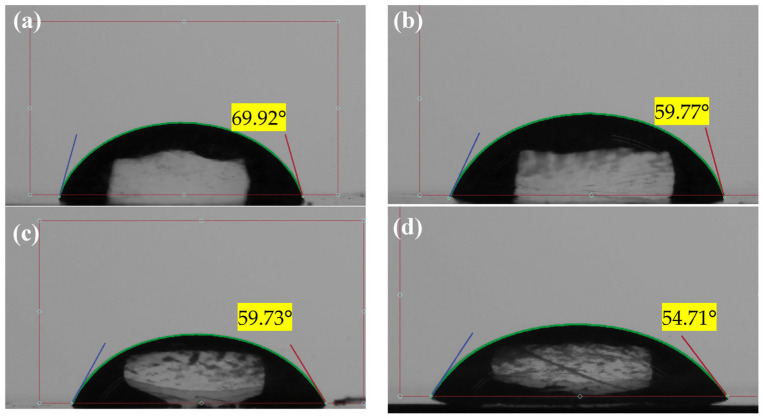
WCA images of the fabricated membranes: (**a**) M0, (**b**) M1, (**c**) M2, and (**d**) M3.

**Figure 4:**
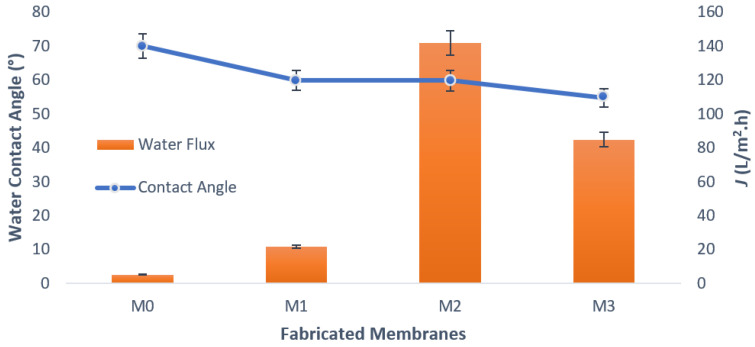
Water contact angle of composite PSf/Ag-GO membranes and pure water flux at applied pressure 7 bar.

The surface wettability played a role in determining the hydrophilicity of a membrane. More hydrophilic membranes have a smaller water contact angle (Mahdi et al., 2019) [48]. The water contact angle (WCA) of pure PSf membranes and nanohybrid membranes was measured using a water contact angle goniometer (DSA100, KrussGmbH, Hamburg, Germany) at room temperature. Images of contact angles for hydrophilicity analysis are presented in Figure 3, whereas Figure 4 displays the value of the water contact angle for each membrane together with water flux values for the composite PSf/Ag-GO membrane. In Figure 3, blue, red, and green line represent left fitted contact angle, right fitted contact angle and droplet width, respectively. The graph in Figure 4 shows that there was a steady drop in WCA from 69.92° to 54.71°. The trend indicates that the WCA of the membrane surface decreased with the addition of Ag-GO NPs. This implies that the membrane became more hydrophilic. This increased hydrophilicity was due to the hydrophilic functional groups of the graphene oxide embedded into the membrane, which may have aided in increasing the water flux of the membrane (Zhang et al., 2013) [49].

According to documented sources, there is a correlation between the hydrophilicity of a membrane and its water flux, where an increase in hydrophilicity results in a corresponding increase in water flux [50]. However, the graph demonstrates that the water flux increased significantly to 141.6 L/m^2^.h for M2 but then decreased to 84.7 L/m^2^.h for M3. The water flux decreased because of the high viscosity of the blended solutions. Mahmoudi et al. [47] found that the addition of a high concentration of GO to the polymer resulted in an increase in solution viscosity. This increase in viscosity led to a reduction in mass transfer between the solvent and non-solvent phases. Rezaee and colleagues also reported that the water flux of the membrane decreased when 0.8 wt% Ag-GO was added. The observed phenomenon can be attributed to the correlation between the rate of pore formation and the rate of solvent and non-solvent exchange in the coagulation bath during the phase inversion process, as previously noted in a study conducted by Rezaee et al. [50]. A higher rate of exchange between the solvent and non-solvent during the coagulation process results in the formation of larger pores. Hence, it can be concluded that the pore size of M3 is smaller in comparison to M2. Overall, the study conducted by Rezaee and colleagues has similarity in terms of the decline in water flux when the amount of Ag-GO is increased.

### 3.3. FESEM Images and EDX Mapping

**Figure 5:**
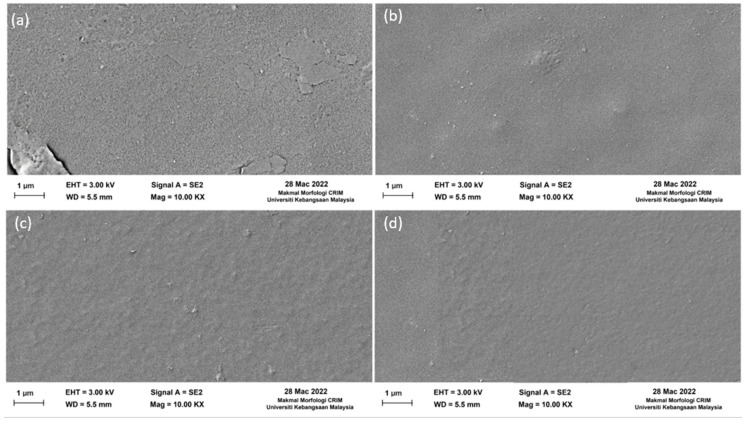
Surface FESEM images of the fabricated membranes: (**a**) M0, (**b**) M1, (**c**) M2, and (**d**) M3.

The surface of the fabricated membranes is depicted in Figure 5 through FESEM images. Based on these images, there is little distinction between the pure PSf membrane (Figure 5a) and the fabricated nanohybrid membrane (Figure 5b–d). The images show no defects or agglomerations on the surface; the surface is smooth and even, with no visible graphene nanoplates as the amount of embedded Ag-GO NPs to the membrane was increased.

The present discovery aligns with an investigation carried out by Zinadini et al. [51], which affirmed the absence of nanoparticle aggregation when employing carbon-based nanofillers. The effective dispersion of nanoparticles in the PSf matrix can be attributed to the chemical composition of graphene oxide, which is carbon-based. However, for non-carbonic particles, such as zinc oxide [52] and titanium dioxide (TiO_2_) [53], there is a significant agglomeration and formation of uneven pore sizes [53]. According to Mataram et al., when 25% TiO_2_ was added to a membrane, there was significant agglomeration and the formation of uneven pore sizes. This is due to nanoparticles migrating to the membranes’ surface [54].

**Figure 6:**
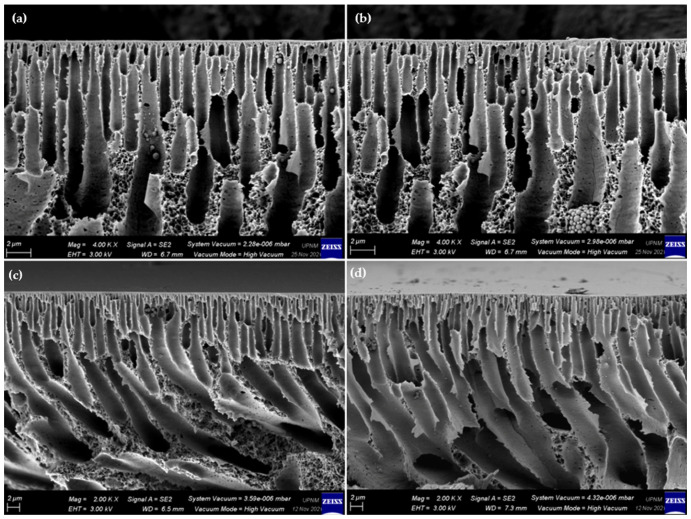
Cross-sectional FESEM images of fabricated membranes: (**a**) M0, (**b**) M1, (**c**) M2, and (**d**) M3.

In contrast with the surface images, the cross-sectional images show a substantial distinction. The morphologies of the prepared PSf membrane and PSf/Ag-GO membranes were investigated via FESEM characterization. Figure 6 shows cross-sectional images of the M0, M1, M2, and M3 membranes. Membranes typically have an asymmetric morphology with finger-like pores linked by sponged walls [55]. However, Figure 6 indicates that the finger-like structure bent slightly as the addition of Ag-GO NPs increased. In addition to that, the bottom part became bigger because GO has high hydrophilicity, attracting additional water during phase inversion. The study conducted by Junaidi and colleagues yielded comparable results. [56].

**Figure 7:**
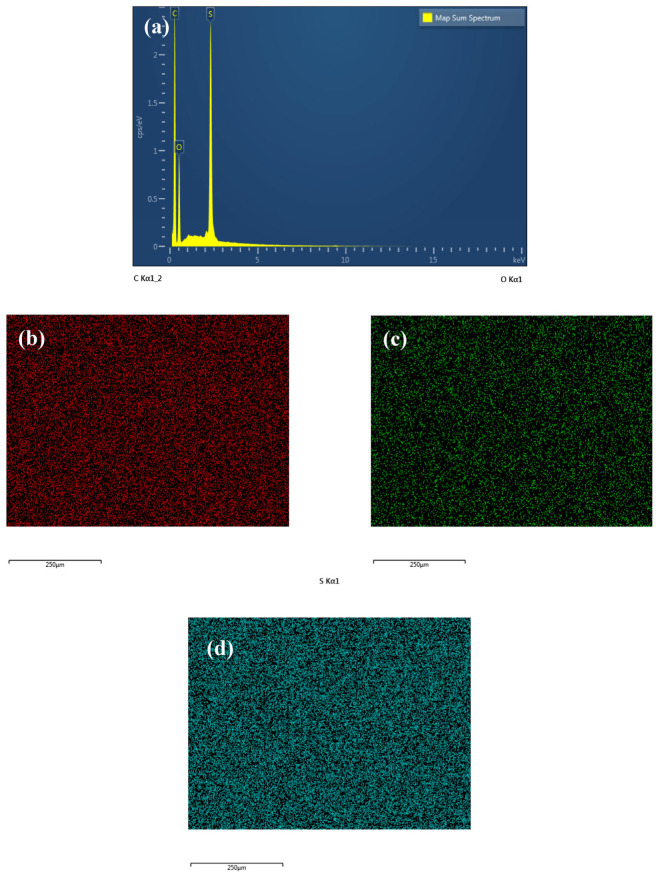
(**a**) EDX spectrum of M0 membrane, (**b**) EDX and FESEM images of carbon, (**c**) oxygen, and (**d**) sulphur mapping.

**Figure 8:**
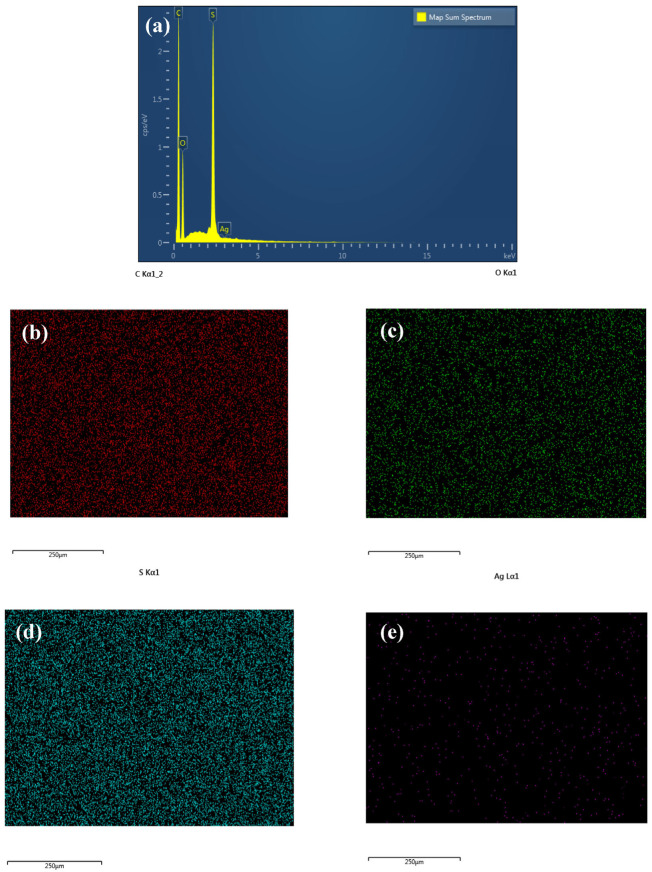
(**a**) EDX spectrum of M2 membrane, (**b**) EDX and FESEM images of carbon, (**c**) oxygen, (**d**) sulphur, and (**e**) silver mapping.

EDX analysis was performed to ascertain and validate the existence of Ag-GO NPs within the mixed matrix of the fabricated membranes. Figure 7 displays the EDX spectrum of the M0 membrane, whereas Figure 8 displays the EDX spectrum of the M2 membrane for a comparative study. The findings indicate the existence of carbon, oxygen, and sulphur in the two membranes. Figure 8 displays an additional silver peak, which suggests the existence of silver nanoparticles within the matrix membrane. In addition, the utilization of the EDX mapping mode was employed to examine the dispersion of silver nanoparticles. From this analysis, silver that was present on the surface of the membrane or within the polyelectrolyte layers was marked with pink dots. Figure 8e shows a lot of pink spots all over the area on the fabricated membrane surface. The above outcome suggests an outstanding dispersion of Ag NPs during the production of the nanohybrid M2 membrane. It is proven that the embedment of Ag NPs reduced the agglomeration of GO in the mixed-matrix membrane.

### 3.4. Salt Rejection

**Figure 9:**
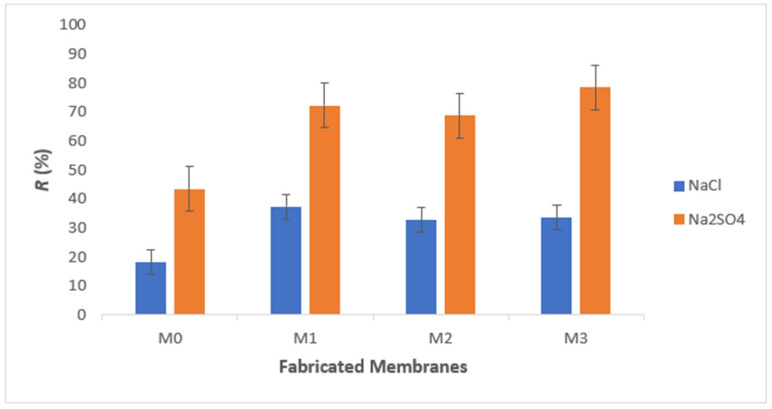
Rejection of salt: NaCl and Na_2_SO_4_ at applied pressure of 7 bar and concentration of 1000 mg L^−1^.

Salt rejection was conducted to gain information about the selective character of membranes. Figure 9 reveals the rejection of sodium chloride (NaCl) and sodium sulphate (Na_2_SO_4_). The nanohybrid membrane exhibited higher salt rejection (78.5%) than the pure PSf membrane (18.2%). These findings were attributed to nanohybrid membranes being more hydrophilic than the pure PSf membranes. Nonetheless, this outcome can also be ascribed to the increasing porosity due to the addition of NPs, in which the porosity of M0 was 68% and M2 was 78%. Based on the graph, the rejection of both salts increased from M0 to M1 but slightly decreased for M2 and then increased again for M3. The observed trend could be ascribed to nanoparticle agglomeration at M2. This agglomeration leads to a reduction in the number of adsorptive active sites and active surface area, ultimately resulting in a decrease in the salt adsorption capacity of the membrane [57].

**Figure 10:**
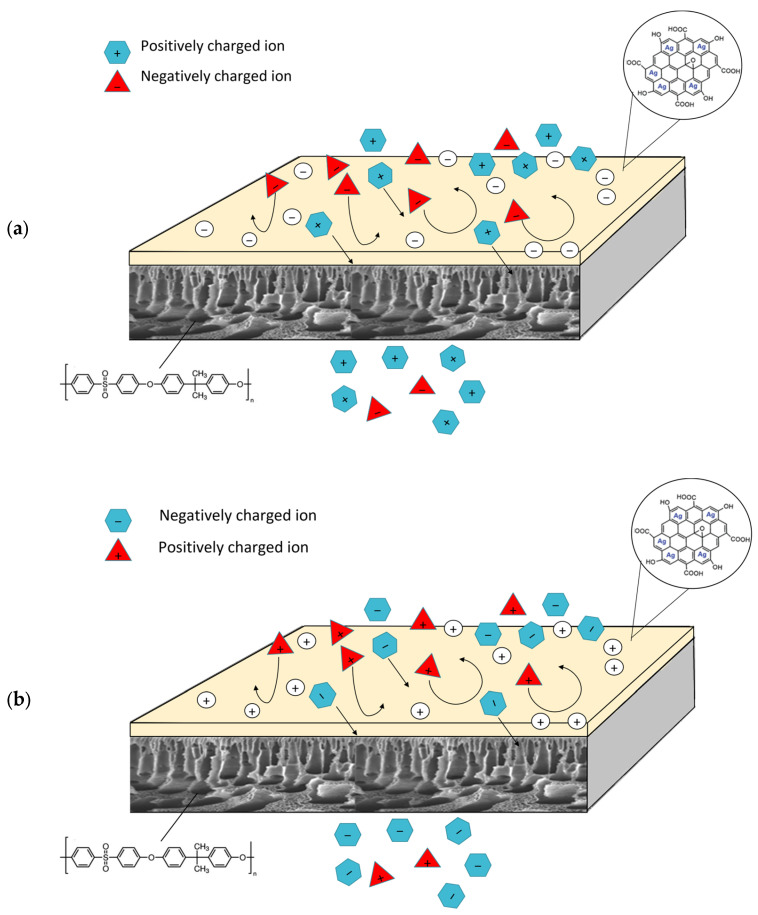
Schematic diagram of Donnan exclusion of (**a**) negatively charged membrane and (**b**) positively charged membrane.

In addition to that, the graph analysis also demonstrates that Na_2_SO_4_ has better rejection for both the pure PSf membrane and nanohybrid membranes compared to NaCl. This is because Donnan exclusion plays an essential role in the rejection mechanism apart from size exclusion. The diagrams depicted in Figure 10a,b demonstrate the mechanism of Donnan exclusion in membranes that are negatively and positively charged, respectively. Membranes with negative charge exhibit selectivity towards positive ions, allowing them to traverse the membrane while repelling negative ions. This process involves the transportation of diverse solutes and the entrapment of certain ions within the channel pore of the membrane. The rejection of ions is enhanced by co-ions with greater charges and reduced by counter-ions with greater charges. The same law is also depicted in positively charged membranes, as shown in Figure 10b. In this study, the co-ions of the negatively charged nanohybrid membrane produced are chloride ions (Cl^−^) and sulphate ions (SO_4_^2−^). In comparison, sulphate ions have higher charges, which explain the high rejection of Na_2_SO_4_ as compared to NaCl. A similar trend was also presented in a study by Seidel et al. [58]. Therefore, these findings demonstrate that the fabricated nanohybrid membrane is suitable for further study on the removal of divalent ions (Fe^2+^), which will be discussed in the next section.

### 3.5. Iron Removal

**Figure 11:**
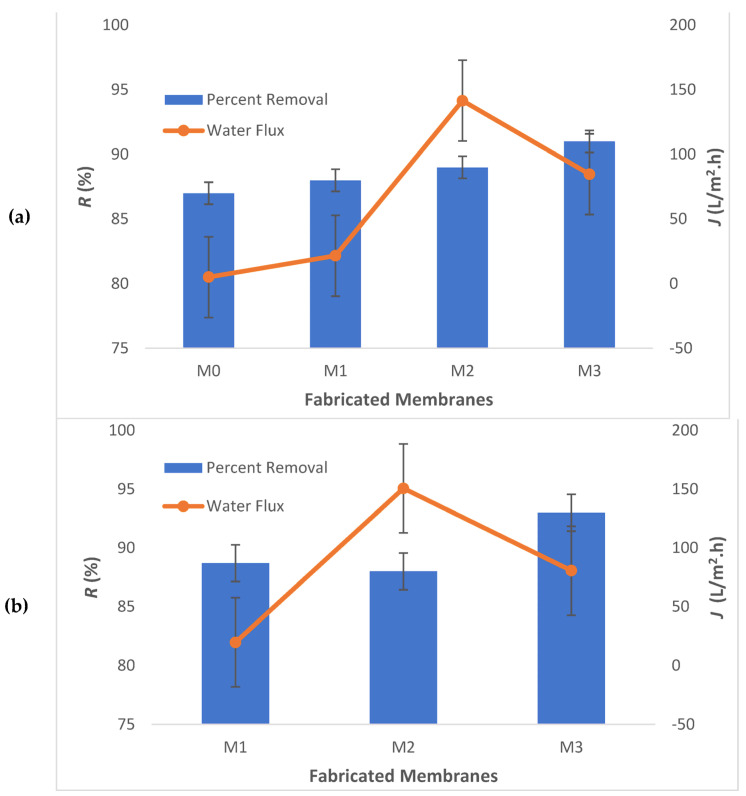
Iron removal and pure water flux of the fabricated membranes: (**a**) feed concentration: 100 mg.L^−1^ and (**b**) feed concentration: 100 mg.L^−1^.

As per the report from the Department of Mineral and Geoscience in Malaysia, the Fe concentrations in naturally occurring groundwater sources were observed to vary between 0.7 and 94 mg L^−1^ [30]. To evaluate the efficiency of the membranes, synthetic groundwater containing various concentrations of Fe^2+^ was prepared. A synthetic groundwater solution was prepared with a concentration of 10 mg L^−1^ for the purpose of investigating the efficacy of the produced membrane at low concentrations. Filtration experiments were carried out under standard laboratory conditions, with an applied pressure of 7 bar and a stirring rate of 300 rpm. The graphical representation depicted in Figure 11 showcases the efficacy of the fabricated membranes in eliminating iron and enhancing water flux. As illustrated in Figure 11a, there was a consistent increase in the percentage of removal. The graph depicts a noteworthy percentage of removal, reaching up to 91%, achieved for the M3 membrane. This suggests that an increase in the weight percentage of graphene oxide in the casting solution led to a corresponding rise in the removal of Fe^2+^ ions.

Then, the filtration experiments were conducted under elevated concentrations. A solution of synthetic groundwater was prepared with a concentration of 100 mg L^−1^ of Fe^2+^. Figure 11b exhibits a comparable pattern to that of Figure 11a. The graph illustrates a marginal rise in the percentage of removal, reaching 93%, when employing the M3 membrane. Owusu-Agyeman et al. [59] made a similar discovery: using an NF membrane to treat river water samples with an elevated concentration of NOM (natural organic matter) of 160 mg L^−1^ led to the removal of more than 90% of NOM. According to the authors, the observed effect can be explained by NOM increasing the charge on the surface of the membrane. Nevertheless, conflicting outcomes were documented; NF90 and NF270 membranes experienced a decrease in Na_2_SO_4_ rejection at extremely high concentrations (15,000 mg L^−1^) [60]. The observed phenomenon can be attributed to the impact of ion deposition on the membrane surface, causing an initial decrease in rejection due to the diffusion of ions, as reported in a study conducted by Kaewsuk et al. [61]. It is reasonable to anticipate that with a much higher concentration of Fe^2+^ ions, there would be a decrease in the percentage of removal.

Currently, the M2 membrane is considered the best membrane in the removal of iron from synthetic groundwater in this study. However, the amount of embedding Ag-GO NPs will be increased in future work to analyze the optimum rejection that can be achieved by these nanohybrid PSf/Ag-GO membranes. The pH of feed solutions for a membrane performance study will also be varied in future work to investigate the possibility of achieving 100% rejection with the aim to reach a safe drinking water level.

## 4. Conclusions

This study successfully synthesized nanohybrid polysulfone membranes embedded with silver decorated with graphene oxide (PSf/AG-GO) by adding Ag-GO NPs to the PSf casting solution. The fabrication of the nanohybrid membrane was achieved through the utilization of the phase inversion method. Due to the incorporation of Ag-GO NPs, the characteristics of the synthesized membrane, such as hydrophilicity, permeability, and selectivity, were improved. This is due to the presence of numerous hydrophilic functional groups on GO. The results of WCA showed that as the amount of embedded Ag-GO NPs is increased, the WCA of the fabricated membrane is decreased, hence showing that the membranes’ hydrophilicity has been enhanced. The permeability of the fabricated membranes also increased since the water flux increased. However, the addition of a higher concentration of GO to the polymeric solution resulted in an incline in the viscosity of the casting solution. This condition, in turn, caused a decrease in mass transfer between the solvent and non-solvent phases. The longer the mass transfer rate, the smaller the pore size produced. Hence, it is recommended to investigate the porosity of the membrane for future work.

Furthermore, the incorporation of graphene oxide (GO) resulted in a slight bending of the finger-like membrane structure, which improved its mechanical strength. Additionally, the presence of GO increased hydrogen bonding, leading to greater water attraction at the bottom of the finger-like structure and ultimately increasing the membrane’s hydrophilicity. Moreover, the energy-dispersive X-ray spectroscopy (EDX) results indicate that the silver oxide nanoparticles were uniformly and densely dispersed on the GO surface, effectively preventing their agglomeration within the membrane matrix.

The selectivity of the synthesized membranes was also increased. The iron removal by the nanohybrid membrane increased to 93% with water flux of 80.59 L/m^2^.h if compared to the pure PSf membrane (88.7% and 19.71 L/m^2^.h). Based on the percent removal, it can be concluded that adding 0.8 wt% of Ag-GO could produce a membrane with extended functionality. However, for future work, it is recommended to investigate whether the M3 membrane is the best membrane amongst all by adding more than 0.8 wt% of Ag-GO NPs. It is also advised to examine the influence of pH on the feed solutions to investigate whether drinking water standards can be reached or if complete removal is possible. The future aim of this work includes further optimization and scale-up of the PSf/Ag-GO membrane for large-scale commercial use, as well as exploring its potential for other applications. For instance, future studies could conduct anti-biofouling tests to investigate the antibacterial properties of the PSf/Ag-GO membrane. Additionally, gas separation and biomedical applications represent exciting avenues for further research and could potentially benefit from the unique properties of the PSf/Ag-GO membrane. Overall, the results of this study offer insights into the design and optimization of nanohybrid membrane systems for more efficient and sustainable processes, and future studies can build upon these findings to develop even more advanced membrane technologies.

## Figures and Tables

**Table 1:** Polysulfone membrane designation based on the polymer-to-nanoplates ratio.

Sample	PSf (wt%)	NMP (wt%)	Ag-GO (wt%)
M0	20	80	0
M1	20	80	0.3
M2	20	80	0.5
M3	20	80	0.8

**Table 2:** Assignment of FTIR spectra of M0 and M2 membranes.

**NO**	Assignment	FTIR Frequency of M0 (cm^−1^)	FTIR Frequency of M2 (cm^−1^)
1	O-H stretching vibrations	3380.84	3382.84 and 3380.84
2	Aromatic C=C stretching	1577.41 and 1485.46	1577.39 and 1485.43
3	C=C	1406.37	1406.20
4	asymmetric O=S=O stretching of sulfone group	1320.34 and 1297.21	1320.22 and 1297.15
5	C-O-C	1238.25	1237.94
6	Symmetric O=S=O	1147.95	1147.79

## Data Availability

Not applicable.

## References

[1] Rusydi A.F., Onodera S.I., Saito M., Ioka S., Maria R., Ridwansyah I., Delinom R.M. (2021). Vulnerability of Groundwater to Iron and Manganese Contamination in the Coastal Alluvial Plain of a Developing Indonesian City. SN Appl. Sci..

[2] Renge V.C., Khedkar S.V., Pande S.V. (2012). Removal of Heavy Metals from Wastewater Using Low Cost Adsorbents: A Review. Sci. Rev. Chem. Commun. J..

[3] Kinuthia G.K., Ngure V., Beti D., Lugalia R., Wangila A., Kamau L. (2020). Levels of Heavy Metals in Wastewater and Soil Samples from Open Drainage Channels in Nairobi, Kenya: Community Health Implication. Sci. Rep..

[4] Engwa G.A., Ferdinand P.U., Nwalo F.N., Unachukwu M.N. (2019). Mechanism and Health Effects of Heavy Metal Toxicity in Humans. Poisoning in the Modern World—New Tricks for an Old Dog?.

[5] Rengel Z. (2004). Heavy Metals as Essential Nutrients. Heavy Metal Stress in Plants.

[6] Usman U.A., Yusoff I., Raoov M., Alias Y., Hodgkinson J., Abdullah N., Hussin N.H. (2021). Natural Sources of Iron and Manganese in Groundwater of the Lower Kelantan River Basin, North-Eastern Coast of Peninsula Malaysia: Water Quality Assessment and an Adsorption-Based Method for Remediation. Environ. Earth Sci..

[7] Khozyem H., Hamdan A., Tantawy A.A., Emam A., Elbadry E. (2019). Distribution and Origin of Iron and Manganese in Groundwater: Case Study, Balat-Teneida Area, El-Dakhla Basin, Egypt. Arab. J. Geosci..

[8] Dvorak B.I., Schuerman B. (2014). Drinking Water: Iron and Manganese. Nebguide.

[9] Crittenden J.C., Trussell R.R., Hand D.W., Howe K., Tchobanoglous G. (2012). Water Treatment: Principles and Design.

[10] Yasin H., Mousa H.M., Abd El-Sadek M.S., Abdel-Jaber G.T. (2020). Membrane Technology for Groundwater Purification: A Review. SVU Int. J. Eng. Sci. Appl..

[11] Kheirieh S., Asghari M., Afsari M. (2018). Application and Modification of Polysulfone Membranes. Rev. Chem. Eng..

[12] Dong L.X., Huang X.C., Wang Z., Yang Z., Wang X.M., Tang C.Y. (2016). A Thin-Film Nanocomposite Nanofiltration Membrane Prepared on a Support with In Situ Embedded Zeolite Nanoparticles. Sep. Purif. Technol..

[13] Changani Z., Razmjou A., Taheri-Kafrani A., Warkiani M.E., Asadnia M. (2020). Surface Modification of Polypropylene Membrane for the Removal of Iodine Using Polydopamine Chemistry. Chemosphere.

[14] Fang L.F., Zhou M.Y., Cheng L., Zhu B.K., Matsuyama H., Zhao S. (2019). Positively Charged Nanofiltration Membrane Based on Cross-Linked Polyvinyl Chloride Copolymer. J. Memb. Sci..

[15] Wang J., Ren Y., Zhang H., Luo J., Woodley J.M., Wan Y. (2021). Targeted Modification of Polyamide Nanofiltration Membrane for Efficient Separation of Monosaccharides and Monovalent Salt. J. Memb. Sci..

[16] Hong Anh Ngo T., Dinh Do K., Thi Tran D. (2017). Surface Modification of Polyamide TFC Membranes via Redox-Initiated Graft Polymerization of Acrylic Acid. J. Appl. Polym. Sci..

[17] Kasim N., Mohammad A.W., Abdullah S.R.S. (2017). Iron and Manganese Removal by Nanofiltration and Ultrafiltration Membranes. Malays. J. Anal. Sci..

[18] Adib H., Raisi A. (2020). Surface Modification of a PES Membrane by Corona Air Plasma-Assisted Grafting of HB-PEG for Separation of Oil-in-Water Emulsions. RSC Adv..

[19] Shen L., Feng S., Li J., Chen J., Li F., Lin H., Yu G. (2017). Surface Modification of Polyvinylidene Fluoride (PVDF) Membrane via Radiation Grafting: Novel Mechanisms Underlying the Interesting Enhanced Membrane Performance. Sci. Rep..

[20] Pendergast M.M., Hoek E.M.V. (2011). A Review of Water Treatment Membrane Nanotechnologies. Energy Env. Sci..

[21] Zakaria Z., Shaari N., Kamarudin S.K., Bahru R., Musa M.T. (2020). A Review of Progressive Advanced Polymer Nanohybrid Membrane in Fuel Cell Application. Int. J. Energy Res..

[22] Mamah S.C., Goh P.S., Ismail A.F., Suzaimi N.D., Yogarathinam L.T., Raji Y.O., El-badawy T.H. (2021). Recent Development in Modification of Polysulfone Membrane for Water Treatment Application. J. Water Process Eng..

[23] Liu M., Zhao L.B., Yu L.Y., Wei Y.M., Xu Z.L. (2020). Structure and Properties of PSf Hollow Fiber Membranes with Different Molecular Weight Hyperbranched Polyester Using Pentaerythritol as Core. Polymers.

[24] Tian J., Chang H., Gao S., Zhang R. (2020). How to Fabricate a Negatively Charged NF Membrane for Heavy Metal Removal via the Interfacial Polymerization between PIP and TMC?. Desalination.

[25] Zhang L., Zhang R., Ji M., Lu Y., Zhu Y., Jin J. (2021). Polyamide Nanofiltration Membrane with High Mono/Divalent Salt Selectivity via Pre-Diffusion Interfacial Polymerization. J. Memb. Sci..

[26] Adamczak M., Kamińska G., Bohdziewicz J. (2019). Preparation of Polymer Membranes by in Situ Interfacial Polymerization. Int. J. Polym. Sci..

[27] Chen B., Jiang H., Liu X., Hu X. (2017). Molecular Insight into Water Desalination across Multilayer Graphene Oxide Membranes. ACS Appl. Mater. Interfaces.

[28] Abu Seman M.N., Hilal N., Khayet M. (2013). UV-Photografting Modification of NF Membrane Surface for NOM Wfouling Reduction. Desalination Water Treat..

[29] Susanto H., Desiriani R., Prasetyo A.A., Hermita D., Istirokhatun T., Nyoman Widiasa I. (2019). Incorporation of Nanoparticles as Antifouling Agents into PES UF Membrane. Mater. Today Proc..

[30] Kasim N., Mahmoudi E., Mohammad A.W., Abdullah S.R.S. (2017). Influence of Feed Concentration and PH on Iron and Manganese Rejection via Nanohybrid Polysulfone/Ag-GO Ultrafiltration Membrane. Desalination Water Treat..

[31] Christian P., Von Der Kammer F., Baalousha M., Hofmann T. (2008). Nanoparticles: Structure, Properties, Preparation and Behaviour in Environmental Media. Ecotoxicology.

[32] Ba-Abbad M.M., Mohammad A.W., Takriff M.S., Rohani R., Mahmoudi E., Faneer K.A., Benamo A. (2017). Synthesis of Iron Oxide Nanoparticles to Enhance Polysulfone Ultrafiltration Membrane Performance for Salt Rejection. Chem. Eng. Trans..

[33] Mahmoudi E., Ng L.Y., Mohammad A.W., Ba-Abbad M.M., Razzaz Z. (2018). Enhancement of Polysulfone Membrane with Integrated ZnO Nanoparticles for the Clarification of Sweetwater. Int. J. Environ. Sci. Technol..

[34] Parvizian F., Ansari F., Bandehali S. (2020). Oleic Acid-Functionalized TiO_2_ Nanoparticles for Fabrication of PES-Based Nanofiltration Membranes. Chem. Eng. Res. Des..

[35] Ponnaiyan P., Nammalvar G. (2020). Enhanced Performance of PSF/PVP Polymer Membrane by Silver Incorporation. Polym. Bull..

[36] Chai P.V., Law J.Y., Mahmoudi E., Mohammad A.W. (2020). Development of Iron Oxide Decorated Graphene Oxide (Fe_3_O_4_/GO) PSf Mixed-Matrix Membrane for Enhanced Antifouling Behavior. J. Water Process Eng..

[37] Pendolino F., Armata N. (2017). Remediation Process by Graphene Oxide. Graphene Oxide in Environmental Remediation Process.

[38] Smith A.T., LaChance A.M., Zeng S., Liu B., Sun L. (2019). Synthesis, Properties, and Applications of Graphene Oxide/Reduced Graphene Oxide and Their Nanocomposites. Nano Mater. Sci..

[39] Cao N., Zhang Y. (2015). Study of Reduced Graphene Oxide Preparation by Hummers’ Method and Related Characterization. J. Nanomater..

[40] Zaaba N.I., Foo K.L., Hashim U., Tan S.J., Liu W.W., Voon C.H. (2017). Synthesis of Graphene Oxide Using Modified Hummers Method: Solvent Influence. Procedia Eng..

[41] Ma C., Hu J., Sun W., Ma Z., Yang W., Wang L., Ran Z., Zhao B., Zhang Z., Zhang H. (2020). Graphene Oxide-Polyethylene Glycol Incorporated PVDF Nanocomposite Ultrafiltration Membrane with Enhanced Hydrophilicity, Permeability, and Antifouling Performance. Chemosphere.

[42] Liu Y. (2017). Application of Graphene Oxide in Water Treatment. IOP Conf. Ser. Earth Env. Sci..

[43] Zhu J., Lua A.C. (2021). Antibacterial Ultrafiltration Membrane with Silver Nanoparticle Impregnation by Interfacial Polymerization for Ballast Water. J. Polym. Sci..

[44] Sprick C.G. (2017). Functionalization of Silver Nanoparticles on Membranes and Its Influence on Biofouling. Master’s Thesis.

[45] Mahmoudi E., Ng L.Y., Ang W.L., Chung Y.T., Rohani R., Mohammad A.W. (2019). Enhancing Morphology and Separation Performance of Polyamide 6,6 Membranes by Minimal Incorporation of Silver Decorated Graphene Oxide Nanoparticles. Sci. Rep..

[46] Bouchareb S., Doufnoune R., Riahi F., Cherif-Silini H., Belbahri L. (2021). High Performance of Polysulfone/Graphene Oxide-Silver Nanocomposites with Excellent Antibacterial Capability for Medical Applications. Mater. Today Commun..

[47] Mahmoudi E. (2017). Silver Decorated Graphene Oxide Embedded Membranes for Enhanced Fouling and Biofouling Control Ebrahim. Ph.D. Thesis.

[48] Mahdi N., Kumar P., Goswami A., Perdicakis B., Shankar K., Sadrzadeh M. (2019). Robust Polymer Nanocomposite Membranes Incorporating Discrete TiO_2_ Nanotubes for Water Treatment. Nanomaterials.

[49] Zhang J., Xu Z., Mai W., Min C., Zhou B., Shan M., Li Y., Yang C., Wang Z., Qian X. (2013). Improved Hydrophilicity, Permeability, Antifouling and Mechanical Performance of PVDF Composite Ultrafiltration Membranes Tailored by Oxidized Low-Dimensional Carbon Nanomaterials. J. Mater. Chem. A.

[50] Rezaee R., Nasseri S., Mahvi A.H., Nabizadeh R., Mousavi S.A., Rashidi A., Jafari A., Nazmara S. (2015). Fabrication and Characterization of a Polysulfone-Graphene Oxide Nanocomposite Membrane for Arsenate Rejection from Water. J. Environ. Health Sci. Eng..

[51] Zinadini S., Zinatizadeh A.A., Rahimi M., Vatanpour V., Zangeneh H. (2014). Preparation of a Novel Antifouling Mixed Matrix PES Membrane by Embedding Graphene Oxide Nanoplates. J. Memb. Sci..

[52] Chung Y.T., Mahmoudi E., Mohammad A.W., Benamor A., Johnson D., Hilal N. (2017). Development of Polysulfone-Nanohybrid Membranes Using ZnO-GO Composite for Enhanced Antifouling and Antibacterial Control. Desalination.

[53] Mataram A., Anisya N., Nadiyah N.A., Afriansyah A. (2020). Fabrication Membrane of Titanium Dioxide (TiO_2_) Blended Polyethersulfone (Pes) and Polyvinilidene Fluoride (Pvdf): Characterization, Mechanical Properties and Water Treatment. Key Eng. Mater..

[54] Badrinezhad L., Ghasemi S., Azizian-Kalandaragh Y., Nematollahzadeh A. (2018). Preparation and Characterization of Polysulfone/Graphene Oxide Nanocomposite Membranes for the Separation of Methylene Blue from Water. Polym. Bull..

[55] Mahmoudi E., Ng L.Y., Ba-Abbad M.M., Mohammad A.W. (2015). Novel Nanohybrid Polysulfone Membrane Embedded with Silver Nanoparticles on Graphene Oxide Nanoplates. Chem. Eng. J..

[56] Junaidi N.F.D., Khalil N.A., Jahari A.F., Shaari N.Z.K., Shahruddin M.Z., Alias N.H., Othman N.H. (2018). Effect of Graphene Oxide (GO) on the Surface Morphology & Hydrophilicity of Polyethersulfone (PES). IOP Conf. Ser. Mater. Sci. Eng..

[57] Bagheripour E., Moghadassi A., Hosseini S.M. (2016). Preparation of Mixed Matrix PES-Based Nanofiltration Membrane Filled with PANI-Co-MWCNT Composite Nanoparticles. Korean J. Chem. Eng..

[58] Seidel A., Waypa J.J., Elimelech M. (2001). Role of Charge (Donnan) Exclusion in Removal of Arsenic from Water by a Negatively Charged Porous Nanofiltration Membrane. Environ. Eng. Sci..

[59] Owusu-Agyeman I., Reinwald M., Jeihanipour A., Schäfer A.I. (2019). Removal of Fluoride and Natural Organic Matter Removal from Natural Tropical Brackish Waters by Nanofiltration/Reverse Osmosis with Varying Water Chemistry. Chemosphere.

[60] Al-Zoubi H., Hilal N., Darwish N.A., Mohammad A.W. (2007). Rejection and Modelling of Sulphate and Potassium Salts by Nanofiltration Membranes: Neural Network and Spiegler-Kedem Model. Desalination.

[61] Kaewsuk J., Lee D.Y., Lee T.S., Seo G.T. (2012). Effect of Ion Composition on Nanofiltration Rejection for Desalination Pretreatment. Desalination Water Treat..

